# Maternal nutrition, inadequate gestational weight gain and birth weight: results from a prospective birth cohort

**DOI:** 10.1186/s12884-016-1012-y

**Published:** 2016-08-15

**Authors:** Anke Diemert, Susanne Lezius, Mirja Pagenkemper, Gudula Hansen, Alina Drozdowska, Kurt Hecher, Petra Arck, Birgit C. Zyriax

**Affiliations:** 1 Department of Obstetrics and Fetal Medicine, University Medical Center Hamburg-Eppendorf, Martinistrasse 52, Hamburg, 20246 Germany; 2 Institute for Medical Biometry and Epidemiology, University Medical Center Hamburg-Eppendorf, Hamburg, 20246 Germany; 3 Preventive Medicine, University Heart Center, University Medical Center Hamburg-Eppendorf, Hamburg, Hamburg

**Keywords:** Pregnancy, Gestational weight gain, Birth cohort, Diet, Nutrient intake, Overweight, Obesity

## Abstract

**Background:**

The aim of our study was to examine maternal weight gain as well as nutrient intake in pregnancy throughout each trimester compared to current recommendations in a low-risk population and its correlation to birth weight. Additionally, we have investigated the association of maternal nutrition with gestational weight gain and birth weight in an economically unrestricted population.

**Methods:**

Our analysis was carried out in a population-based prospective birth cohort in Hamburg, Germany. 200 pregnant women and 197 infants born at term were included in the analysis. Maternal body weight, weight gain throughout gestation, and birth weight, macro- and micronutrients were assessed based on a 24 h dietary recall in each trimester. Our main outcome measures were weight gain, birth weight, and self-reported dietary intake in each trimester in comparison to current recommendations.

**Results:**

One third of the women were characterized by an elevated pre-pregnancy BMI, 60 % did not comply with current weight gain recommendations. Particularly overweight and obese women gained more weight than recommended. In a multivariate analysis birth weight correlated significantly with maternal BMI (*p* = 0.020), total weight gain (*p* = 0.020) and gestational week (*p* < 0.001). Compared to guidelines mean percentage of energy derived from fat (*p* = 0.002) and protein (*p* < 0.001) was significantly higher, whereas carbohydrate (*p* = 0.033) intake was lower. Mean fiber intake was significantly lower (*p* < 0.001). Saturated fat and sugar contributed largely to energy consumption. Gestational weight gain correlated significantly with energy (*p* = 0.027), carbohydrates (*p* = 0.008), monosaccharides and saccharose (*p* = 0.006) intake. 98 % of the pregnant women were below the iodine recommendation, while none of the women reached the required folate, vitamin D, and iron intake.

**Conclusions:**

During gestation appropriate individual advice as to nutrient intake and weight gain seems to be of high priority. Pregnancy should be used as a ‘window of opportunity’ for behavioral changes.

## Background

An increased body-mass-index (BMI) at the beginning of pregnancy and inadequate gestational weight gain (GWG) represent a substantial health burden for mother and child. Normal weight for women at childbearing age should be strived for. However, the global obesity epidemic has resulted in an increasing number of overweight and obese women entering pregnancy. Data from cross-sectional studies indicate that at present about 20 % of the German women aged 20–39 are overweight, another 9–14 % are obese and up to 18 % are affected by central obesity [1, 2]. In addition a substantial proportion of pregnant women seems to be characterized by an inadequate GWG according to the recommendations of the US Institute of Medicine (IOM) [3, 4]. Both extremes, excessive or inadequate GWG can lead to adverse pregnancy outcomes. Maternal complications arising from excessive GWG include an increased rate of caesarean section, hypertensive disorders, diabetes, weight retention and abdominal adiposity. The latter conditions determine a women’s lifetime risk for cardiovascular disease [5–11]. Fetal risks includes stillbirth, miscarriage, malformation, macrosomia, as well as an increased risk of premature death and trans-generational obesity [12–17].

Adherence to a balanced diet throughout pregnancy, influences maternal body weight as well as short- and long-term health of mother and child [18–20]. Data of the ‘German National Nutrition Survey II’ indicate, that average nutrient intake in women of childbearing age is at least in parts inadequate [21]. During gestation the need for certain micronutrients increases more than energy requirement [22]. Thus, deficiencies in micronutrients may occur frequently, leading to adverse outcomes for mother and child. However, information as to dietary intake throughout gestation is limited [23, 24]. Most birth cohorts did not evaluate maternal dietary habits. Nutritional data for pregnant women derive mainly from cross-sectional studies where pregnant women represent a minority. Furthermore, in previous studies nutrient intake was measured only once during pregnancy. Considering the fact that pregnancy constitutes a unique opportunity to change dietary behavior in order to preserve maternal and fetal health more research on this topic is warranted.

The aim of this paper was therefore to evaluate maternal weight gain and nutrient intake throughout pregnancy in an economically unrestricted prospective birth cohort comprising mostly normal pregnancies and to compare the nutrient intake to current guidelines and birth weight. Additionally, we have investigated the association of maternal nutrition with gestational weight gain and birth weight.

## Methods

### Design and Recruitment

From 2011–2013 200 healthy low risk women aged 18 years and older with singleton spontaneously conceived pregnancies presented at the University medical Centre Hamburg-Eppendorf to participate in this study between gestational age 12 + 0 to 14 + 6 weeks. Exclusion criteria were risk factors according to menstrual- (such as unknown last menstrual period), obstetrical- (such as history of IUGR or preeclampsia) and medical history (other diseases), drug usage (such as alcohol or tobacco) or chronic medication.

At 3 time points (once per trimester (gestational week 12 + 0 to 14 + 6, 22 + 0 to 24 + 6 and 34 + 0 to 36 + 6) participants presented for a detailed fetal ultrasound examination, psychometric and lifestyle questionnaires (24 hours-recall) and maternal blood draw. Additionally a morphology scan was performed at gestational week 20–24 and birth weight and pregnancy outcome was collected. Sociodemographic data such as familial status and education were also reported to ensure no constraints regarding dietary options.

### Data collection

#### Assessment of anthropometric data

The maternal height and weight - light clothing, but no shoes allowed - were measured to the nearest 0.5 cm or 0.1 kg respectively, and taken with the same instruments (scale and yardstick) that were calibrated in regular intervals for the whole population at each visit. The body mass index (BMI) was calculated as BMI = weight (kg)/height (m)^2^.

Pregnancy weight gain was compared with current S3 guideline on gestational diabetes derivated from the recommendation of the US Institute of Medicine (IOM), depending on BMI classes: 12.5–18 kg for underweight women, 11.5–16 kg for normal weight women, 7–11.5 kg for overweight women and 5–9 kg for obese women [25, 26].

#### Nutritional counseling and assessment of nutrition

Pregnant women included in our study were counseled on guideline recommended nutrition during pregnancy by physicians certified for nutritional medicine. Information about nutrition was obtained by using a 24 h dietary recall in each trimester based on the nutritional software EBISpro System based on the national food composition table (BLS 3.01). Recommendations for dietary intake of energy, macro- and micronutrients during pregnancy were based on the D-A-CH reference value of the German, Swiss and Austrian society for nutrition [22].

### Data analysis/Statistical methods

Continuous variables are presented as means (± standard deviations). Categorical variables are presented as absolute numbers and rates. Mean nutrition values are derived by patient-individual averaging.

Comparisons to guidelines or recommendations are performed using the one-sample *t*-test.

Time trends were determined with random effects models allowing for autoregression with a random intercept for the individual patients and adjusted for education level und maternal age. Associations with maternal weight gain and birth weight were analyzed using ANOVA models adjusted for maternal age, maternal BMI at the beginning of pregnancy, education level and in case of birth weight additionally for gestational week. Further adjustments are stated separately.

Not normally distributed parameters were log transformed where necessary. All analyses were two-sided with α = 0.05. No alpha adjustment for multiple testing was applied.

All calculations are performed using SPSS Version 21 (SPSS Inc., Cary, NC, USA).

## Results

### Baseline characteristics

Baseline characteristics of the study population are listed in Table 1. All women were married or living in a stable relationship. The study population consisted of 191 Caucasian, seven Asian, one African and one Latin American women. 78 % of the participants had a high educational level and two-thirds a normal weight at the beginning of pregnancy. One third were overweight (defined as BMI > =25 but lower than 30) or obese (defined as BMI > =30). 57 % of study subjects were primipara.

**Table 1:** Baseline characteristics of the PRINCE birth cohort. Depicted is either the mean value or the percentage value. Values are given as mean ± 1 standard deviation (SD) or as absolute and relative frequencies (^a^ *n* = 197, ^b^ *n* = 196)

Characteristics	Total *n* = 200
Clinical characteristics	
Maternal age (years)	31.0 (±3.5)
Height (cm)	168 (±6)^a^
*Weight*	
Weight at the beginning of pregnancy (kg)	69.9 (±13.9)^a^
BMI (kg/m^2^)	24.7 (4.6)^a^
BMI <18,5 (kg/m^2^)	4 % (*n* = 8)
BMI 18,5 - <25 (kg/m^2^)	63 % (*n* = 124)
BMI 25 - <30 (kg/m^2^)	21 % (*n* = 41)
BMI ≥30 (kg/m^2^)	12 % (*n* = 24)
*Parity*	
First pregnancy	57 % (*n* = 114)
Former pregnancy	43 % (*n* = 86)
1 living child	49 % (*n* = 41)
≥2 living children	13 % (*n* = 11)
*Delivery*	
Gestational age at delivery (weeks)	40.0 (±1.5)^a^
Fetal weight at delivery (g)	3457 (±497)^b^
<3rd percentile	2 % (*n* = 3)
>97th percentile	2 % (*n* = 4)
Life birth	197 (98 %)
Pregnancy discontinued (termination, miscarriage)	3 (2 %)
Lifestyle characteristics	
*Education level* ^b^	
No high school diploma	22 % (*n* = 44)
High school or vocational diploma	34 % (*n* = 66)
College or higher education	44 % (*n* = 86)
*Smoking habits*	
Current non-smokers	100 %
*Alcohol use*	
Alcohol abstinence	100 %

### Energy and macronutrient intake

During gestation the average daily energy intake in our study population increased from 1987 kcal (±505) in the first trimester to 2068 kcal (±463) in the second and 2151 kcal (±472) in the last trimester (*p* = 0.001 for increase between first and 3rd trimester). Protein intake increased significantly from 76 g (±24) in the first trimester to 80 g (±21) in the second (*p* = 0.037) and 82 g (±23) in the third trimester (*p* = 0.003 for increase between first and 3rd trimester). Major sources for protein were animal products (data not shown). With regard to the fat consumption daily intake increased significantly from 77 g (±29) to 82 g (±27) and 86 g (±28) over the trimesters (*p* = 0.003 for increase between first and 3rd trimester). Saturated fatty acids (SAFA) contributed mainly to mean total fat consumption (SAFA = 36 g ±10 of total fat 81 g ± 19), whereas the intake of monounsaturated fatty acids (26 g ± 7) and polyunsaturated fatty acids (13 g ±4) was much lower. Mean intake of docosahexaenoic acid (DHA), derivated from marine sources was 221 mg (±243). Throughout gestation carbohydrate intake raised from 239 g (±65) in the first trimester to 245 g (±66) in the 2nd trimester to 254 g (±66) in the last trimester non-significantly (*p* = 0.152). Monosaccharide and saccharose contributed largely to the carbohydrate consumption (43 %), while mean fiber intake was low (24 g ±6). When additionally adjusted for total energy no significant changes in macronutrient intake throughout pregnancy were observed.

If macronutrient intake was calculated in percentage of energy intake this was 16 % from protein, 36 % from fat, and 49 % from carbohydrates. In comparison to current recommendations the overall mean protein and fat intake of the participants was significantly higher than recommended (protein 16 vs. 15 %, *p* <0.001 and fat 36 vs. 35 %, *p* = 0.002). In addition, the percentage of saturated fat intake with regard to total fat consumption was much higher than recommended (45 % vs. 10 %, *p* <0.001), whereas mean carbohydrate intake was significantly lower than recommended (49 vs. 50 %, *p* = 0.033) (DACH Referenzwerte, 2015).

When categories instead of mean values were taken into account 38 % of the pregnant women reported a protein intake below the recommended 15 % of energy intake. Within the study population 58 % of the pregnant women reported a fat intake above the upper threshold of 35 % of energy predominately characterized by a high consumption of saturated fat, while 62 % of the women did not fulfill the criteria of a sufficient intake of docosahexaenoic acid. As to carbohydrate intake 56 % of the women were below the recommendation of 50 % of energy. The percentage of women consuming less than 30 g fiber per day was 84 %. In each trimester mean dietary intake of protein, fat, carbohydrates and fiber differed significantly from the recommendation with the exception of fat and carbohydrate intake during the first trimenon.

### Micronutrient intake

Mean intake of vitamins and minerals from food increased slightly between the first and the last trimester (Table 2). However, most changes were not significant, particularly when additionally adjusted for energy intake (data not shown). In each trimester mean dietary intake of certain micronutrients differ significantly from the recommendation (Table 2).

**Table 2:** Intake of micronutrients during pregnancy compared with current recommendations (DACH Referenzwerte, 2015) [22]. Values are given as mean ± 1 standard deviation (SD)

Critical-micronutrients	1. trimenon	2. trimenon	3. trimenon	mean intake	recommendation	*p*-value
Iron (mg)	12 ± 4	13 ± 3	12 ± 3	12 ± 2	30	<0.001
Iodine (μg)	115 ± 62	130 ± 86	123 ± 67	123 ± 48	230	<0.001
Vit D (μg)	2.5 ± 3.7	2.4 ± 2.3	2.8 ± 4.4	2.6 ± 2.1	20	<0.001
Folat (μg)	273 ± 124	291 ± 119	296 ± 133	287 ± 81	550	<0.001
-Other-micronutrients						
Vit A (mg)	1.4 ± 1.7	1.7 ± 1.5	1.5 ± 1.4	1.5 ± 0.9	1.1	<0.001
Vit E (mg)	13 ± 7	14 ± 7	14 ± 8	13.5 ± 5.0	13	n.s.
Vit B1 (mg)	1.3 ± 0.5	1.4 ± 0.6	1.4 ± 0.6	1.3 ± 0.4	1.3	n.s.
Vit B2 (mg)	1.5 ± 0.7	1.7 ± 0.6	1.8 ± 0.7	1.7 ± 0.4	1.4	<0.001
Vit B6 (mg)	1.7 ± 0.7	1.8 ± 0.7	1.9 ± 0.8	1.8 ± 0.5	1.9	0.002
Vit B12 (μg)	4.7 ± 4.7	4.6 ± 2.3	4.9 ± 2.7	4.7 ± 2.2	3.5	<0.001
Vit C (mg)	146 ± 90	158 ± 94	166 ± 105	158 ± 67	105	<0.001
Calcium (mg)	1133 ± 487	1193 ± 457	1227 ± 502	1179 ± 324	1000	<0.001
Magnesium (mg)	359 ± 103	388 ± 99	386 ± 106	378 ± 76	310	<0.001
Zinc (mg)	11 ± 4	12 ± 3	11 ± 3	11 ± 2	10	<0.001

#### Vitamins

**Fig. 1:**
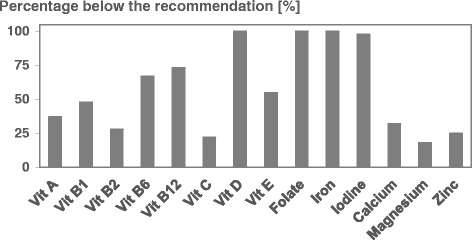
Percentage of women below the recommended dietary intake of vitamins and minerals (DACH Referenzwerte, 2015) [22]

Compared to current recommendations mean dietary intake of vitamin A, E, B_1_, B_2_, and B_12_ seems to be adequate (Table 2) [22]. However, on the average a considerable amount of the women did not reach the recommendation on vitamin A (37 %), vitamin E (55 %), vitamin B_1_ (48 %), vitamin B_2_ (28 %) and vitamin B_12_ (73 %) intake (Fig. 1). In addition 23 % of the pregnant women did not reach the recommended intake of vitamin C, while 67 % reported an insufficient intake of vitamin B_6_ (Fig. 1). With regard to folate and vitamin D mean daily intake from food was much lower than recommended (folate 288 μg (±126) vs 550 μg, *p* < 0.001; vitamin D 2.6 μg (±3.6) vs 20 μg, *p* < 0.001) (Table 2). (DACH Referenzwerte, 2015). None of the pregnant women fulfilled on average the recommendation as to folate or vitamin D intake (Fig. 1).

#### Minerals

An iron requirement of 30 mg throughout pregnancy is recommended, whereas mean daily iron intake in the study population was only 12 mg (±2 *p* < 0.001) (Table 2) (DACH Referenzwerte, 2015). Thus, in 100 % of the pregnant women iron supply in each trimester was inadequate (Fig. 1). For iodine the mean daily intake was 123 μg (±48) which is significantly less than the recommendation of 230 μg (*p* < 0.001) (Table 2) (DACH Referenzwerte, 2015). On the average 98 % of the women were below the recommended threshold (Fig. 1).

Mean daily intake of calcium, magnesium and zinc seems to be adequate in comparison to the recommendation (calcium 1179 mg (±324) vs 1000 mg; magnesium 378 mg (±76) vs 310 mg, zinc 11 mg (±2) vs 10 mg). However, as to calcium, 32 % of the pregnant women ingested less than recommended, while 18 % of the women were below the recommendation of magnesium and 26 % below the recommended zinc intake (Fig. 1).

Besides the intake of micronutrients from food, 96 % (*n* = 192) of the women reported to use supplements. However, evaluation of the obtained data is difficult as various products were reported and most supplements were not used continuously (data not published).

### Maternal BMI and gestational weight gain

Mean maternal BMI at the beginning of pregnancy was 24.7 (±4.6), with 63 % of the women being normal weight, 4 % being underweight, 21 % overweight and 12 % obese at this time. In the second trimester maternal weight reached a BMI of 26.7 (±4.6), while in the third trimester mean BMI was 28.5 (±4.5).

Mean maternal weight gain in our study cohort was 11.2 kg (±3.9). Compared to current guidelines only 40 % of our pregnant women gained weight during gestation appropriately, 22 % more than recommended and 38 % less than recommended. Particularly underweight and overweight women did not follow gestational weight gain recommendations. In a multivariate model no association could be determined between GWG and maternal age, education, parity or fetal sex, whereas pre-pregnancy BMI (*p* = 0.003) was a significant predictor for the amount of weight gained during pregnancy

**Fig. 2:**
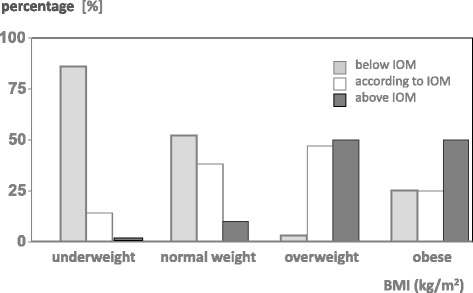
Gestational weight gain according to the recommendation of the Institute of Medicine (IOM) (Institute of Medicine, 2009) [4]

Within each BMI categories significant deviations from the recommendation were observed. According to maternal BMI at the beginning of pregnancy 86 % of the underweight women gained less weight than suggested, whereas only 14 % gained within the recommended range (Fig. 2). Of the normal weight participants every second woman (52 %) gained less than recommended, 38 % appropriate and only a small minority (10 %) above the recommendation. The opposite distribution was noted in the overweight population: every second woman (50 %) gained weight above the recommendation, 47 % according to the recommendation, whereas in 3 % gestational weight gain was insufficient. In obese women 50 % gained above and 50 % according to the recommendation.

In separate analyses total gestational weight gain correlated significantly with energy intake (*p* = 0.036). Per each 100 cal increment maternal weight increased by 195 g (95 % CI 13–378). A positive association was also found, when carbohydrate intake or consumption of monosaccharide and saccharose was taken into account. An increase of 1 g carbohydrate was related to an increment of 17 g in weight during pregnancy (95 % CI 5–29; *p* = 0.008), while 1 g sugar was associated with an increase of 26 g weight (95 % CI 8–44; *p* = 0.005). After further adjustment for energy intake, the correlation between sugar consumption and gestational weight gain remained significant (*p* = 0.049) whereas the association between carbohydrates and weight gain lost significance (*p* = 0.097). After adjustment for energy intake other macronutrients or fiber intake were not associated with gestational weight gain.

### Birth weight

Two women terminated the pregnancy due to fetal malformations and one aborted spontaneously. Of the 197 births (birth weight was available in *n* = 196 cases), mean birth weight was 3457 g (±497), 24 (12 %) of the children were characterized by a birth weight > 4000 g. Newborn‘s weight was lower in normal weight mothers compared to overweight, obese and underweight mothers. In a multivariate analysis birth weight correlated significantly with maternal BMI at the beginning of pregnancy (*p* = 0.020), total weight gain (*p* = 0.020) and gestational week (*p* < 0.001) For each unit increment in pre-pregnancy BMI birth weight increased by 17 g (95 % CI 3–32), whereas each kilogram weight gained during pregnancy leads to an increase in birth weight by 20 g (95 % CI 3–36). An increase in birth weight by 138 g was observed per gestational week (95 % CI 89–187). All the other nutrients tested in this analysis were not associated significantly with birth weight.

## Discussion

### Main Findings

The aim of this analysis was to evaluate maternal weight gain as well as nutrient intake throughout each trimester of pregnancy compared to international recommendations and birth weight in a low risk population. Currently information as to weight development and dietary habits in pregnant women is limited.

A principal finding of our prospective birth cohort is that approximately two third of pregnant women did not comply regarding to gestational weight gain to current guidelines [4]. This has been previously described for other countries [26]. Every second woman with overweight or obesity and more than one third of the normal weight women gained more weight than recommended, whereas the majority of underweight women failed to gain sufficient weight. After adjustment for education, maternal age, baseline BMI and duration of the pregnancy a significant correlation between total weight gain and birth weight persisted

In addition a substantial proportion of women were characterized by poor dietary habits throughout the whole pregnancy although our population represents a rather educated (78 % higher education) and socioeconomically unrestricted population, which are factors associated with a better diet. As to macronutrients, more than every second women reported a fat consumption above the recommendation, characterized by a high intake of saturated fat, while the intake of polyunsaturated fatty acids, namely docosahexaenoic acid was insufficient. Compared to current guidelines the consumption of carbohydrates and fiber was mostly too low, and nearly half of the carbohydrates were obtained from sugar. Energy, as well as carbohydrate and sugar intake correlated significantly with total gestational weight gain even after adjustment for education and maternal age. The observed association between weight gain and monosaccharides and saccharose consumption remained significant even after further adjustment for energy intake.

With regard to micronutrient requirement a sustainable number of pregnant women fail to comply with the recommended daily intake. On the average none of the women reached the recommendation as to iron, folate and vitamin D and almost all participants reported an iodine intake below the guideline were independent from education levels or BMI categories.

### Strengths and Limitations

To our knowledge our study is one of the first population–based ones evaluating not only gestational weight gain but also detailed dietary habits in a sample of German pregnant women. Data was collected prospectively, whereas most existing birth cohorts have recruited women in the course of pregnancy. Mean maternal age and rates of overweight and obesity are representative of the German population. Weight and nutrient intake was assessed in each trimester and compared to current recommendations.

However there are limitations that need to be addressed. First, our findings are confined to those women who voluntarily chose to take part in this study. Second, the sample is rather small and consisted of married women with a higher educational level. Third, we recorded maternal weight at the beginning of pregnancy in the first trimester. This did not differ significantly to recorded pre-pregnancy weight gain in our cohort (data not shown) and is widely used for these analysis as a true pre-pregnancy weight can only be obtained by patient recall. However we could draw the conclusion that in fact pregnancy weight gain could be even higher. Finally, nutrient assessment was obtained from only three 24 h recalls per women, one in each trimester. Further research is needed to confirm our results. In spite of these limitations, our data provides information as to weight gain and nutrient intake throughout pregnancy, findings that will have important implications for clinical practice.

### Interpretation

Mean maternal age in the PRINCE cohort is representative of the German population (mean maternal age at delivery of the first child is 30.2 years in Germany [27]. One third of the women were characterized by overweight or obesity, which is in line with findings from the German National Nutrition Survey II and the German DEGS-Study in women of childbearing age [1, 2]. Some studies reported, that maternal weight at the beginning of pregnancy seems to have a greater impact on health of mother and child than weight gained throughout pregnancy [19, 28]. However, inadequate gestational weight gain, particularly excessive weight gain is known to be associated with unfavorable pregnancy outcomes [7–9, 11]. In line with most previous research birth weight correlated significantly with maternal BMI at the beginning of pregnancy as well as with weight gain and gestational week even after further adjustment for education and age in a multivariate model. However most of the existing studies did not take into account gestational week. Excessive weight gain can be used as a potential predictor for offspring’s overweight and obesity, particularly in normal weight women [3].

In addition, potential priming effects of high maternal weight gain on offsprings’s overweight cannot be excluded [29]. Mean total gestational weight gain in our study was slightly below data reported from two other population-based birth cohorts [3]. Consistent with other studies two thirds of our cohort did not follow recommendations as to weight gain [3]. Weight development throughout pregnancy should also be discussed in the light of maternal post-partum weight retention, further pregnancies and lifelong cardiovascular risk for the mother. Women whose doctors recommended weight gains consistent with IOM guidelines were more likely to follow the recommendation [30]. Results from intervention studies indicate that nutrition and lifestyle counseling usually combined with supplementary weight monitoring in pregnant women reduced the rate of pregnancies with excessive gestational and weight retention at six months postpartum weight gain without increasing insufficient weight gain and seems to be safe [31–33].

Dietary habits before and throughout pregnancy influence short- and long-term health of mother and child over and beyond a potential influence on gestational weight gain. Particularly a low intake in critical micronutrients such as folate, iodine or iron leads to fetal complications. Maternal dietay habits may even play a role in terms of in utero programming of offspring appetite [34] and food preference of the offspring [35]. Up to now detailed information as to energy intake, macronutrients and micronutrient content of the diet at different stages of gestation are limited [36]. In our study population the mean energy intake increases significantly by 8 % during the course of pregnancy, which is in line with the recommended 10 % [22]. However, the individual energy need may vary substantially depending mainly on the level of physical activity. Experience has shown, that most women decrease their physical activity throughout gestation, leading to the current recommendation that additional calories in overweight and obese women should be avoided [22].

More than one out of ten women did not reach the recommended protein intake in our study, while every second women was above the recommended fat intake throughout pregnancy. Data from animal studies indicate that high fat maternal diets during pregnancy seems to have adverse effects in offspring with regard to exercise performance, hepatic lipid accumulation, insulin resistance, and development of atherosclerosis [37–39]. As to carbohydrate and fiber intake the majority of women were obviously below the recommendation.

Present findings reflect in most parts mean dietary intake of macronutrients and fiber in the women of childbearing age in the German population, leading to the assumption, that without targeted intervention “healthy” pregnant women will not change the quality of their diet [1]. Initiatives to promote a healthy lifestyle during pregnancy like the German ‘Healthy Start – Young Family Network’ are helpful to increase awareness, however, more individual advice is highly recommended [19]. A mismatch between dietary practice and macronutrient recommendation in pregnant women is supported by a systematic review and meta-analysis including data of developed countries [23]. The quality of fat and carbohydrate intake is also of increasing interest. In our study saturated fat contributed largely to the fat consumption, while the intake of polyunsaturated fatty acids, namely docosahexaenic acid was too low. These findings are in line with findings from other European countries [23]. Carbohydrate intake, particularly sugar seems to be an important determinate of gestational weight gain in our cohort, which is supported by data from a Danish birth cohort, analyzing the impact of added sugar on excessive weight development during pregnancy [40].

To supply adequate amounts of vitamins, minerals and other micronutrients a nutrient-dense diet is desirable, particularly in the light of an increase in the requirement of certain micronutrients. Consistent with a meta-analysis in developed countries, our findings indicate that pregnant women are at risk of suboptimal micronutrient intake [24]. On the average none of the women met the recommendation as to folate, iron, and vitamin D throughout pregnancy and almost all were below the recommendation as to iodine intake. These findings reflect in most parts mean dietary intake of vitamins and minerals in women of childbearing age in the German population. The reference values for folate, iron, iodine and vitamin D can hardly be reached through diet alone [1, 22]. Supplying micronutrients to protect deficiencies can be useful to reduce adverse outcomes, but should not replace a healthy well-balanced diet. In addition, further studies to evaluate the effect of various combination and doses of micronutrients are warranted [41].

## Conclusions

In summary, our analysis contributes to the limited literature on nutrient intake and weight gain throughout pregnancy. The data clearly indicate that the majority of women do not follow current recommendations as to dietary habits and gestational weight gain. Increasing rates of obese women of childbearing age will strengthen the problem. In the light of the present study a lifestyle counseling delivered to all pregnant women is highly justified, as intervention during pregnancy are characterized by a unique treatment adherence and can prevent short-term and long-term health risk of mother and child. Dietary advice is part of the prenatal care in Germany. However, this is mostly done by gynecologists and not by nutritional experts. Often this information is given as a leaflet to the patients. This highlights the need of high quality nutritional brochures but also individual advice affect the metabolic and weight trajectory both for women and their offspring.

## Abbreviations

BMI, body-mass-index; D-A-CH, Germany-Austria-Switzerland; DHA, docosahexaenoic acid; GWG, gestational weight gain; IOM, US Institute of Medicine; IUGR, intra-uterine growth restriction; SAFA, Saturated fatty acids; SPSS, Statistical Package for the Social Sciences
